# Dynamic variation of the microbial community structure during the long-time mono-fermentation of maize and sugar beet silage

**DOI:** 10.1111/1751-7915.12263

**Published:** 2015-02-25

**Authors:** Johanna Klang, Susanne Theuerl, Ulrich Szewzyk, Markus Huth, Rainer Tölle, Michael Klocke

**Affiliations:** 1 Department Bioengineering, Leibniz Institute for Agricultural EngineeringMax Eyth Allee 100, Potsdam, 14469, Germany; 2 Department of Environmental Microbiology, Technische Universität BerlinErnst-Reuter-Platz 1, Berlin, 10587, Germany; 3 Department of Crop and Animal Sciences, Humboldt-Universtät zu BerlinUnter den Linden 6, Berlin, 10099, Germany

## Abstract

This study investigated the development of the microbial community during a long-term (337 days) anaerobic digestion of maize and sugar beet silage, two feedstocks that significantly differ in their chemical composition. For the characterization of the microbial dynamics, the community profiling method terminal restriction fragment length polymorphism (TRFLP) in combination with a cloning-sequencing approach was applied.

Our results revealed a specific adaptation of the microbial community to the supplied feedstocks. Based on the high amount of complex compounds, the anaerobic conversion rate of maize silage was slightly lower compared with the sugar beet silage. It was demonstrated that members from the phylum *Bacteroidetes* are mainly involved in the degradation of low molecular weight substances such as sugar, ethanol and acetate, the main compounds of the sugar beet silage. It was further shown that species of the genus *Methanosaeta* are highly sensitive against sudden stress situations such as a strong decrease in the ammonium nitrogen (NH_4_^+^-N) concentration or a drop of the pH value. In both cases, a functional compensation by members of the genera *Methanoculleus* and/or *Methanosarcina* was detected. However, the overall biomass conversion of both feedstocks proceeded efficiently as a steady state between acid production and consumption was recorded, which further resulted in an equal biogas yield.

## Introduction

One important objective for the future energy supply worldwide is to disengage from the dependence on fossil fuels and nuclear energy and instead extend the use of renewable energy sources. In this context, the production of biogas, containing energy-rich methane, is one important technique for energy production. Biogas is produced through the anaerobic digestion (AD) of organic matter, e.g. energy crops and animal manure. The production of biogas is unique among renewable energies, because it is suitable for the simultaneous production of electricity and heat, as a fuel and as a substitute for natural gas (FNR, 2013). In addition, the production of biogas is independent of daily and seasonal as well as weather-related fluctuations. Therefore, this technology, or more precisely, this process can be used for securing the basic supply of electricity. For the last years, there has been an increased cultivation of energy crops in Germany, which are used as feedstock for the production of energy-rich biogas (Balussou *et al*., 2012), whereby maize accounts for the largest share (FNR, 2012). Maize offers several advances as feedstock for the AD, including a high amount of dry matter and a high potential biogas yield as well as low requirements of fertilization and plant protection products during cultivation. On the other hand, there is an increasing criticism concerning the cultivation of maize caused by negative influences on soil fertility and biodiversity. As a consequence, the research efforts for alternative feedstocks, for example sugar beet, which have a similar potential to maize in terms of the resulting biogas yield, are intensified (FNR, 2013).

The conversion of biomass into biogas is an anaerobic process mediated by a complex microbial community. The process can roughly be divided into four phases: hydrolysis, acidogenesis, acetogenesis and methanogenesis. In the first phase, particular organic polymers, such as carbohydrates, lipids and proteins, are hydrolyzed into sugars, fatty acids and amino acids, which are further degraded into the intermediates volatile fatty acids (VFAs), acetate, alcohols, carbon dioxide (CO_2_) and hydrogen (H_2_) during the acidogenesis and the acetogenesis. In the last phase, methane (CH_4_) is produced either from acetate (acetoclastic) or from hydrogen and carbon dioxide (hydrogenotrophic) (Gujer and Zehnder, 1983).

The first three phases are conducted by organisms from the domain *Bacteria*, whereby the phyla *Firmicutes*, *Bacteroidetes*, *Proteobacteria* and *Chloriflexi* are the most abundant ones (Nelson *et al*., 2011). The last phase, the methanogenesis, is performed by representatives from the domain *Archaea* where the three main orders *Methanobacteriales*, *Methanomicrobiales* (both hydrogenotrophic) and *Methanosarcinales* are prevalently found (Nelson *et al*., 2011). The latter order can be divided into the obligate acetoclastic family *Methanosaetaceae* and the mixotrophic family *Methanosarcinaceae*, which are the physiological generalist among the *Archaea* as they can switch, depending on prevailing conditions, between the two main metabolic pathways (Liu and Whitman, 2008).

In order to investigate the high community complexity in regard of the composition and dynamic of the process-involved microorganisms, different molecular biological methods are frequently applied (Klocke *et al*., 2007; Wang *et al*., 2010; Carballa *et al*., 2011; Regueiro *et al*., 2012; Fotidis *et al*., 2014). The most commonly used methods, for example cloning/sequence, quantitative “real-time” polymerase chain reaction (qPCR) or community profiling techniques like the terminal restriction fragment length polymorphism (TRFLP) or the denaturing gradient gel electrophoresis (DGGE), are based on analyses of the 16S rRNA gene. This gene is well known for its high phylogenetic resolution power for detection of microbial relationships. A combination of different methods and a correlation with the process parameters enable a complementary and comprehensive investigation that allows to link community structure information to its phenotypic role in its respective habitat.

In this study a comparative investigation between the mono-fermentation of maize and sugar beet silage was performed. It was assumed that maize silage will be characterized by a diminished biodegradability due to a higher amount of organic polymers resulting in a higher structural and functional microbial diversity. In comparison with this, sugar beet silage has a higher amount of easy degradable compounds that probably favours the development of secondary degraders. Therefore, our research objective was to investigate the specific community adaptation due to differences in the chemical compositions of the energy crops as well as the community changes in a long-term experiment of 337 days during mono-fermentation in regard to possible nutrient deficits.

## Results and discussion

### Biogas production kinetics of maize and sugar beet mono-fermentation

The analysis of the investigated feedstocks maize silage and sugar beet silage showed great differences in their chemical composition (Table 1). The total solid (TS) was around two times higher in the maize silage compared with the sugar beet silage. However, the maize silage had higher amounts of complex polymeric compounds such as lignin, cellulose, hemicellulose, starch, crude fat and crude protein, whereas the sugar beet silage contained more easy degradable compounds such as sugar, ethanol and acetate. The complex compounds have to undergo all four process phases before biogas is produced, whereby the hydrolysis is considered to be the rate-limiting step (Pavlostathis and Giraldo-Gomez, 1991). In contrast to that, the compounds of the sugar beet silage can more or less be directly converted into biogas.

**Table 1:** Chemical composition of the supplied feedstocks maize silage (MS) and sugar beet silage (SBS) as well as the ratio of each compound in comparison of MS and SBS

Parameter	Unit	MS	SBS	MS:SBS
TS	(% FM)	27	14	2:1
VS	(% TS)	96	95	1:1
Lignin	(g kg_FM_^−1^)	6	4 × 10^−2^	142:1
Cellulose	(g kg_FM_^−1^)	58	5	12:1
Hemicellulose	(g kg_FM_^−1^)	49	4	14:1
Starch	(g kg_FM_^−1^)	0.8	0.2	4:1
Sugar	(g kg_FM_^−1^)	0.01	0.17	1:19
Crude fat	(g kg_FM_^−1^)	1.0 × 10^−4^	5.3 × 10^−6^	20:1
Crude protein	(g kg_FM_^−1^)	20	7	3:1
TKN	(g kg_FM_^−1^)	3.2	1.3	2.5:1
NH_4_^+^-N	(g kg_FM_^−1^)	0.06	0.11	1:2
Ethanol	(g kg_FM_^−1^)	2	24	1:12
Acetate	(g kg_FM_^−1^)	5	10	1:2

Values are given as single measurements of a composite sample. TS = total solids, FM = fresh mass, VS = volatile solids, TKN = total Kjeldahl nitrogen, NH_4_^+^-N = ammonium nitrogen.

**Figure 1:**
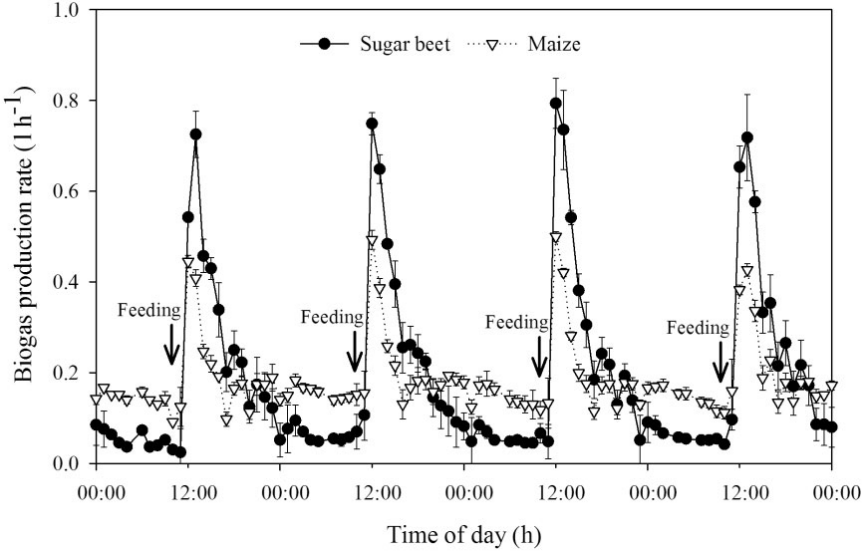
Highly temporal resolution of the kinetics of the biogas production rates over 4 days at OLR 2.0 g_VS_ l^−1^ day^−1^ for both the anaerobic digestion of maize silage and sugar beet silage. Shown are mean values including the standard deviation of the three parallel reactors per feedstock. “Feeding” indicate time-points of substrate addition.

For each feedstock, three parallel continuously stirred tank reactors (CSTRs) with a working volume of 3 l were operated at mesophilic conditions (40°C). The mentioned differences in the chemical composition of the feedstocks are reflected in the kinetics of the biogas production (Fig. 1). Shortly after feeding, the easy degradable compounds were degraded resulting in the maximum biogas production in both reactor systems, whereby the sugar beet reactors yielded higher biogas production rates than the maize reactors. After this first consumption/conversion phase, only more complex compounds such as starch, hemicellulose and cellulose were available for microbial degradation. As the maize silage was characterized by a higher amount of these compounds, here the biogas production was higher over time, whereas only a low biogas production was found in the sugar beet reactors around 12 h after feedstock addition. Nevertheless, the mean biogas yield was equal for both feedstocks with 0.64 ± 0.02 l_N_ g_VS_^−1^ day^−1^ (with 51% CH_4_) for maize silage and 0.67 ± 0.01 l_N_ g_VS_^−1^ day^−1^ (with 54% CH_4_) for sugar beet silage, which is in agreement with substrate-specific biogas yields previously published by KTBL (2009).

### Reactor performance and process efficiency

All reactors were inoculated with digestate from an agricultural biogas plant feed with a mixture of different energy crops and animal manure. Compared with the inoculum, the results showed a decrease in the TS and volatile solids (VS) of the digestates for both systems after the changeover from the feedstock mixture to the sole substrates maize and sugar beet silage (Table S1). During the subsequent experimental phase, an enrichment of the TS and VS was recorded in the maize reactor systems, which was accompanied with a decrease in the degradation degree of VS from 90 ± 0.1% at day 33 to 78 ± 0.3% at day 337. In contrast, the degradation degree of VS in the sugar beet system was rather constant over the entire trail period with 87 ± 0.6%. Also these differences may be explained by the differences in the chemical composition of the feedstocks as the lower degradation degree in the maize reactors is caused by the high amount of complex compounds of the supplied feedstock. The hydrolyzable compounds hemicellulose and cellulose of the maize plant material are protected from biodegradation as the anaerobically non-degradable lignin is forming a matrix that surrounds the (hemi-) cellulose microfibrils (Kirk and Farrell, 1987; Ress *et al*., 1998), resulting in a diminished biodegradability and hence a lower degradation rate. However, the overall degradation degree was slightly higher, but in a general agreement with practical experiences where the mean VS degradation degree is reported with 76% (FNR, 2010). It can be assumed that the overall process proceeded efficiently and that there was a steady state between acid production and consumption as no VFA accumulation was recorded (Table S1).

**Figure 2:**
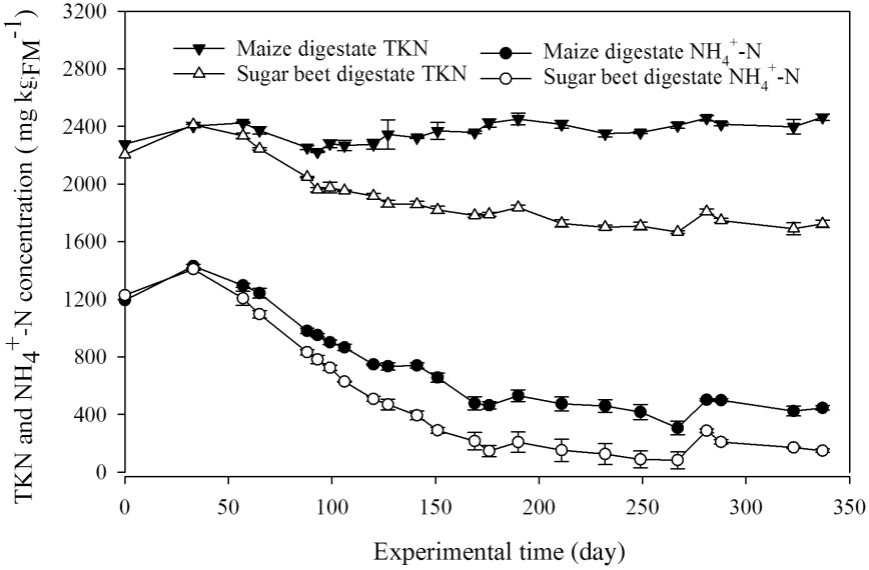
Total Kjeldahl nitrogen (TKN) and ammonium nitrogen (NH_4_^+^-N) concentration of the maize reactor digestate as well as the sugar beet reactor digestate over the entire experimental time as mean values including standard deviation of the three parallel reactors per feedstock.

The total Kjeldahl nitrogen (TKN) was rather constant in the maize reactors, but decreased in the sugar beet reactors until around day 141. These findings indicate that the sugar beet reactors required more time to reach stationary conditions caused by the generally lower TKN in the sugar beet silage (Fig. 2). On the other hand, the NH_4_^+^-N concentration constantly decreased in both reactor systems (Fig. 2). Considering that no TKN was accumulated, this indicates that only low protein degradation took place. It can be assumed that the present microorganisms utilized the more easy accessible carbon compounds as primary energy source whereby the proteins were only degraded into amino acids, which were used for their cell growth but not mineralized to ammonium nitrogen. Several studies confirmed that the protein degradation capacity decreased when high amounts of sugar are present (Breure *et al*., 1986; Tommaso *et al*., 2003). This seems to be the case especially in the sugar beet reactors.

### Long-time adaptation of the bacterial communities to different feedstocks

For each feedstock digestion experiment, three parallel CSTRs were operated either with maize silage or sugar beet silage. The TRFLP results are given as median values of the three parallel-operated CSTRs per feedstock digestion experiment. To ensure a high functionality of the initial starter-community (Wittebolle *et al*., 2009), the inoculum used for the start-up consisted of a microbial community specifically adapted to the digestion of a diverse mixture of energy crops and animal manure.

**Figure 3:**
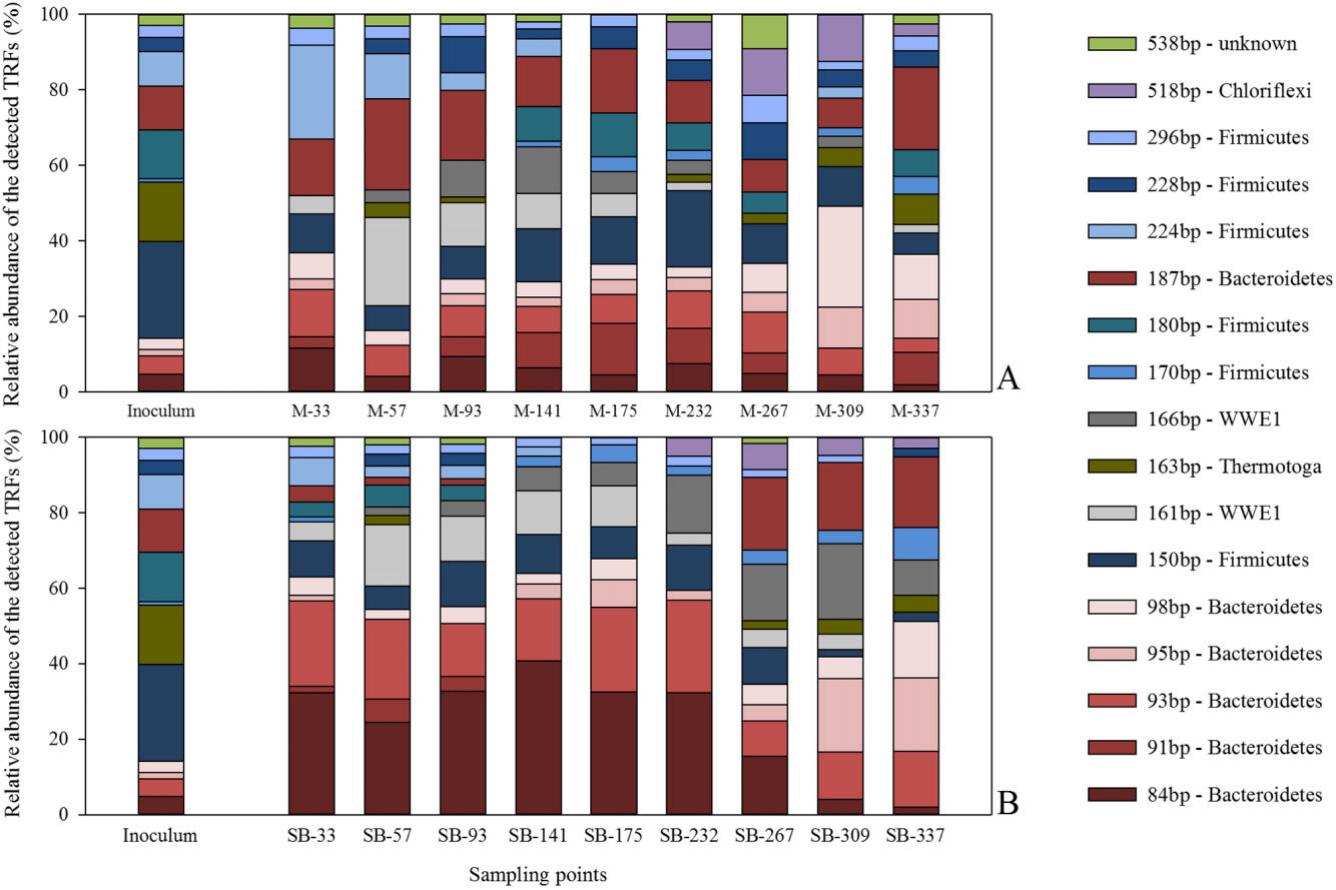
Structure of the bacterial community involved in the biomethanation process of (A) maize silage and (B) sugar beet silage. Shown is the relative abundance of the detected terminal restriction fragments (TRFs) as a function of the percental fluorescence intensity of each individual TRF in relation to the total fluorescence intensity. Coloured bars symbolize TRFs in base pairs (bp), which were identified by 16S rRNA gene sequence libraries. Only TRFs with a relative abundance over 5 % in at least one sample are shown. Each sampling point is given as median value of three biological replicates (i.e*.* parallel-operated biogas reactors) and three technical replicates (i.e. three DNA extracts per reactor). Numbers in sampling point descriptors indicate the duration of continuous fermentation in days.

The results of this study showed that the bacterial community structure is directly influenced by the supplied feedstocks, as an adaptation of the bacterial starter-community was found already at day 33, the end of the first organic loading rate (OLR) stage (Fig. 3). The calculated pairwise distance between these two sampling points (‘inoculum’ and ‘day 33’), considering both the changes in the number as well as the relative abundance of each detected terminal restriction fragment (TRF), showed a change in the community structure of 49% in the maize reactors and 64% in the sugar beet reactors. The community organization became more even (Table S2), meaning that the bacterial community members became more equally distributed in their relative abundance (Verstraete *et al*., 2007; Marzorati *et al*., 2008; Read *et al*., 2011). The three most abundant TRFs from the starter-community (TRF-150bp, TRF-163bp and TRF-180bp) decreased while for example TRF-224bp (family *Ruminococcaceae*) in the maize reactors or TRF-84bp and TRF-93bp (both related to the family *Phorphyromondaceaea*) in the sugar beet reactors gained importance (Fig. 3, Table 2).

**Table 2:** Phylogenetic assignment of the detected bacterial TRFs by screening of 16S rRNA gene sequence libraries using the RDP Classifier as well as selected species examples from the identified families and their physiological potential to identify the functionality of the dominant groups within the maize and sugar beet reactors

Phylogenetic assignment (phylum, class, order, family)	Species examples and their potential function within the AD process chain		Reference
*Bacteroidetes, Bacteroidia, Bacteroidales, Porphymonadaceae*	*Palidibacter propionicigenes*	Sugar fermentation	Ueki and colleagues (2006)
(TRF-84bp, TRF-93bp, TRF-95bp)	*Petrimonas sulfuriphila*	Mono- and disaccharide fermentation	Grabowski and colleagues (2005)
	*Proteiniphilum acetatigenes*	Protein degradation	Chen and Dong (2005)
*Bacteroidetes, Bacteroidia, Bacteroidales, Prevotellaceae* (TRF-98bp)	*Prevotella ruminicola*	Utilization of starch, non-cellulosic polysaccharides, and simple sugars	Purushe and colleagues (2010)
*Fibrobacteres, Fibrobacteria, Fibrobacterales, Fibrobacteraceae* (TRF-150bp)	*Fibrobacter succinogenes*	Only cellulose is hydrolyzed and metabolized; removes xylose-rich hemicelluloses to gain access to cellulose	Suen and colleagues (2011a)
*Firmicutes, Clostridia, Clostridiales, Ruminococcaceae* (TRF-224bp)	*Saccharofermentans acetigenes*	Utilization of starch, non-cellulosic polysaccharides, and simple sugars	Chen and colleagues (2010)
	*Clostridium sufflavum*	Degradation of mono- and disaccharides, xylane and cellulose, but no starch	Nishiyama and colleagues (2009)
	*Ruminococcus albus*	Highly cellulolytic, degrade cellulose and hemicellulose	Suen and colleagues (2011b)
*Firmicutes, Clostridia, Clostridiales, Lachnospiraceae* (TRF-296bp)	*Cellulosilyticum ruminicola*	Degradation of cellulose, hemicellulose and pectin	Cai and colleagues (2010)
	*Lachnospira pectinoschiza*	Pectin degradation	Cornick and colleagues (1994)
	*Clostridium xylanovorans*	Mono- and disaccharide fermentation, non-cellulolytic, non-acido./acetogenic	Mechichi and colleagues (1999)
*Chloriflexi, Anaerolineae, Anaerolineales, Anaerolineaceae* (TRF-518bp)	*Levilinea saccharolytica*	Utilization of monosaccharides, peptides and amino acids and pyruvate	Yamada and colleagues (2006)
	*Longilinea arvoryzae*	Fermentation of divers carbohydrates (incl. hemicellulose) and proteins;	Yamada and colleagues (2007)
		enhanced growth in co-cultivation with hydrogenotrophic methanogens	
WWE1 candidate division	*‘*Candidatus *Cloacamonas acidaminovorans’*	Fermentation of amino acids, sugars, and carboxylic acids; synthrophic	Pelletier and colleagues (2008)
Unknown *Bacteroidetes* (TRF-91bp, TRF-187bp)	No functional relation possible		
Unknown *Firmicutes* (TRF-150bp, TRF-170bp, TRF-180bp)			
Unknown *Thermotogae* (TRF-163bp)			
Unknown *Bacteria* (TRF-161bp, TRF-228bp, TRF-538bp)	No phylogenetic and no functional relation possible		

In comparison with the more even (Gini coefficient of 0.42 ± 0.01) but highly diverse (richness of 48 ± 8) and dynamic bacterial community of the maize reactors (Table S2, Fig. 3A), the bacterial community in the sugar beet reactors remained rather stable as TRF-84bp and TRF-93bp were predominant with a relative abundance between 15–28% and 9–17% until day 232 (Table S2, Fig. 3B). As the NH_4_^+^-N concentration in the reactors reached the minimum values between day 232 and 267 a community change (dissimilarity) of around 30% was recorded by the calculated pairwise distance between these two sampling points, resulting in a community re-organization: in the maize system, TRF-98bp (family *Prevotellaceae*, phylum *Bacteroidetes*) was predominant with an abundance of 17% followed by TRF-518bp (family *Anaerolineaceae*, phylum *Cloriflexi*), TRF-95bp (family *Porphyromonadaceae*, phylum *Bacteroidetes*) and TRF-150bp (class *Clostridia*, phylum *Firmicutes*) with 7–8%. In contrast to that, the former most dominant TRF in the sugar beet reactors (TRF-84bp) decrease to less than 3%, while TRF-187bp (order *Bacteriodales*), TRF-166bp (WWE1 candidate division), TRF-98bp and TRF-95bp gained importance each with up to 12%.

To conclude, these findings showed that the present bacterial community in the maize reactors consisted of a more even distribution of different phyla, whereby the sugar beet reactors were dominated by members of the phylum *Bacteroidetes* with abundances of up to 47%. In contrast to the sugar beet silage, which showed a very narrow substrate spectrum, the digestion of maize silage provided a wide range of metabolites whereby each requires a specific conversion pathway. As the bacterial community in the maize reactors has to be able to perform a successive and complementary biomass conversion with a functional redundancy among diverse phylogenetic groups (Table 2), it is not surprising that the maize reactor systems showed a higher bacterial diversity and higher dynamic variations over time then the sugar beet reactor systems. The high abundance of *Bacteroidetes* within the sugar beet reactors indicates that these microorganisms hold an important part as secondary degrader in the second and third step of the biomass conversion chain, which is in accordance with previously published studies (Ito *et al*., 2012; Hanreich *et al*., 2013).

### Long-time adaptation of the archaeal communities to different feedstocks

As expected, the archaeal population structure revealed a lower diversity than the bacterial one in both reactor systems with a richness of 6 ± 2 and 7 ± 2 TRFs in the maize and sugar beet system, compared with 48 ± 8 and 41 ± 9 TRFs in the bacterial community (Table S2). These findings have previously been reported several times, e.g. by Liu and colleagues (2009), Carballa and colleagues (2011) and Regueiro and colleagues (2012). This is also in agreement with the fact that *Bacteria* are involved in the first three steps of biomass transformation with a high variety of substrates whereby *Archaea* are restricted to a very narrow nutrient spectrum in terms of acetate, methyl-group containing compounds as well as CO_2_ and H_2_.

**Figure 4:**
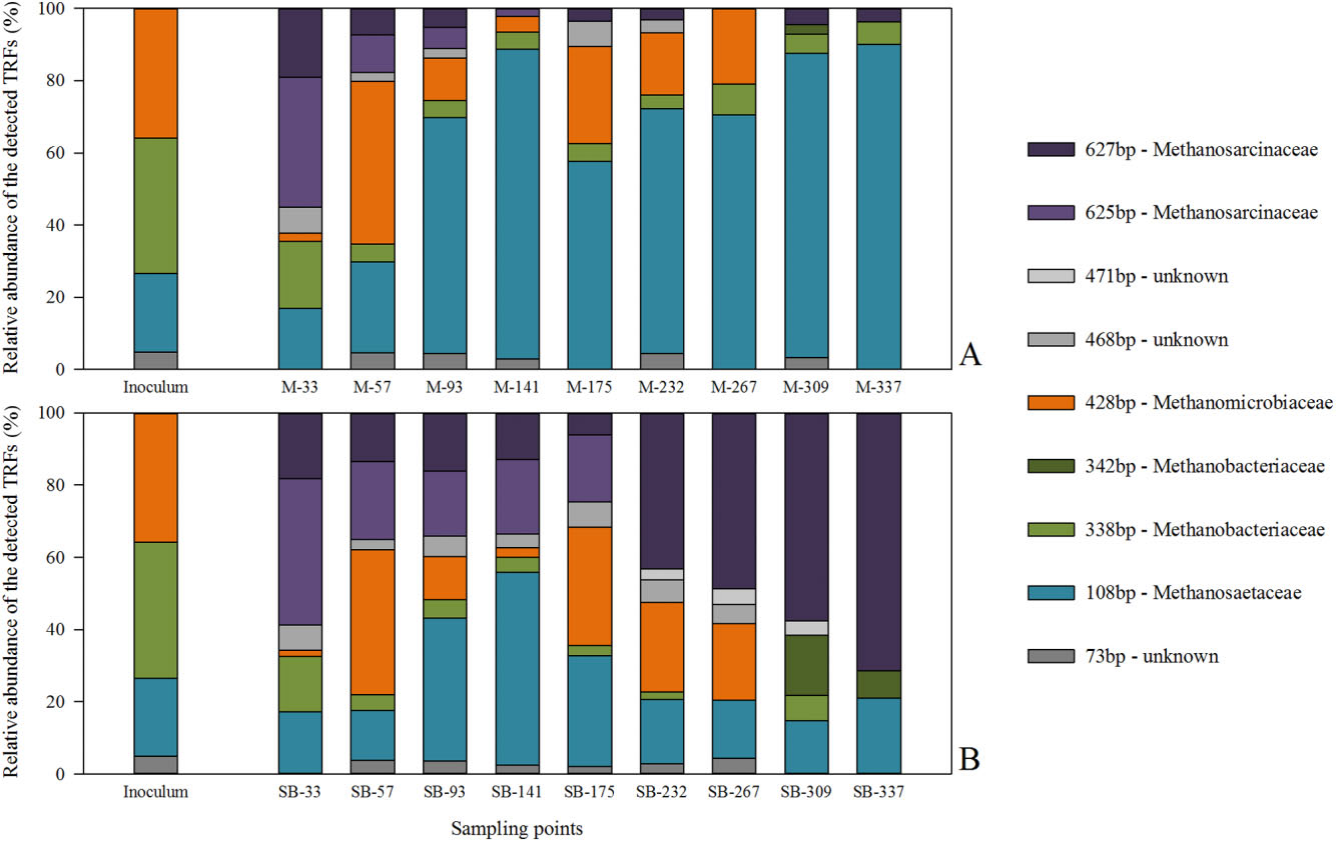
Structure of the archaeal community involved in the biomethanation process of (A) maize silage and (B) sugar beet silage. Shown is the relative abundance of the detected terminal restriction fragments (TRFs) as a function of the percental fluorescence intensity of each individual TRF in relation to the total fluorescence intensity. Coloured bars symbolize TRFs in base pairs (bp), which were identified by 16S rRNA gene sequence libraries. Each sampling point is given as median value of three biological replicates (i.e. parallel-operated biogas reactors) and three technical replicates (i.e. three DNA extracts per reactor). Numbers in sampling point descriptors indicate duration of continuous fermentation in days.

Similar to the bacterial community, the results showed an adaptation of the archaeal starter-community to the supplied feedstocks already at day 33 (Fig. 4): the inoculum was dominated by hydrogenotrophic *Archaea* (represented by TRF-338bp/*Methanobacterium* sp. and TRF-428bp/*Methanoculleus* sp.), caused by the rather high VFA and NH_4_^+^-N concentrations, which are known to inhibit the obligate acetoclastic genus *Methanosaeta* (Schnürer *et al*., 1999; Karakashev *et al*., 2005). After 33 days of reactor performance, the hydrogeonotrophic methanogens were replaced by members of the most versatile (mixotrophic) and stress-tolerant methanogenic family *Methanosarcinaceae* (Liu and Whitman, 2008; De Vrieze *et al*., 2012), more precisely by the genus *Methanosarcina* (represented by TRF-625bp and TRF-627bp), which were under the detection limit within the starter-community. This evident shift in the archaeal community indicates that the reactors, or better, the occurring communities, had to deal with a sudden stress, the feedstock change, and adapted to the new conditions (De Vrieze *et al*., 2012).

**Figure 5:**
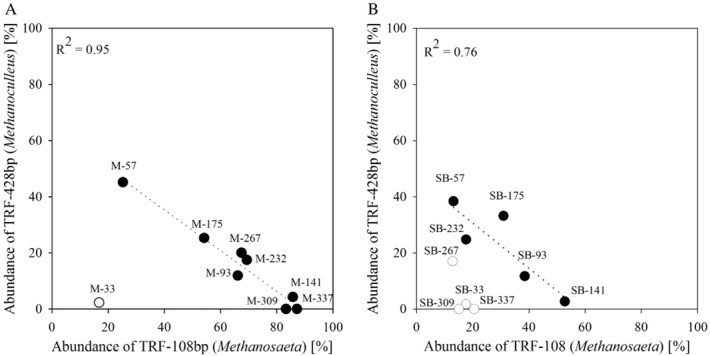
Correlation between the abundance of TRF-428bp (related to *Methanoculleus*) and TRF-108bp (related to *Methanosaeta*) in (A) the maize reactors and (B) the sugar beet reactors. Only samples indicated by a full black dot were considered for correlation. *R*^2^ is the correlation coefficient.

From day 33 to day 57, the calculated dissimilarity values still showed high structural changes between the communities. The changes decreased evidently over time until the community shift reached 1% in the maize system and 5% in the sugar beet system between day 93 and 141, meaning that the archeal community structure became more and more similar over time. During the start-up phase, which was accompanied by a decrease in the NH_4_^+^-N level (Fig. 2), the genus *Methanosaeta*, represented by the TRF-108bp, became predominant with 86% of the total archaeal community in the maize reactors, whereas the abundance of the genus *Methanoculleus* decreased. This can be explained as the inhibitory effect of high NH_4_^+^-N concentration on the acetoclastic methanogenes pathway (e.g. Schnürer and Nordberg, 2008; Fotidis *et al*., 2014) was successively reduced over time. Interestingly and first described in this study, a further decrease of the NH_4_^+^-N concentration promoted the re-occurrence of the genus *Methanoculleus* (between day 175 and 267, Fig. 4A). After day 267 until the end of the experimental phase, the NH_4_^+^-N concentration in the maize reactors was kept rather constant at around 450 mg kg_FM_^−1^. Consequently, the *Methanoculleus*-related TRF-428bp completely disappeared, and finally, the archaeal community of the maize reactor was clearly dominated by the obligate acetoclastic genus *Methanosaeta* (TRF-108bp, 87%). Thus, the results revealed an antagonistic behaviour between the genera *Methanosaeta* (symbolizes by TRF-108bp) and *Methanoculleus* (TRF-428bp), in the maize reactor systems (Fig. 5A). Apparently, species of the genus *Methanosaeta* are highly sensitive against sudden stress situations, whereby *Methanoculleus* seems to be more robust. Moreover, it can be supposed that the decreasing and especially the low NH_4_^+^-N concentration influence not only the archaeal community composition, but also the bacterial one. This in turn may lead to the production of metabolites, which favour the presence of *Methanoculleus*. For example, between day 175 and 267, members from family *Anaerolineacea* (phylum *Cloriflexi*) became abundant (Fig. 3A, Table 2). It is known that the growth for example of the genus *Longilinea* is enhanced in co-culture with hydrogenotrophic methanogens (Yamada *et al*., 2007). Nevertheless, after keeping the NH_4_^+^-N concentration rather constant to avoid a process failure, the genus *Methanosaeta* is dominating the archaeal community again.

Similar results were found for the sugar beet reactors, although the relative abundance of both genera *Methanosaeta* and *Methanoculleus* were lower compared with the maize reactors (Fig. 4B). A significant antagonistic behaviour between *Methanosaeta* and *Methanoculleus*, which is already discussed above, was recorded until day 232 (Fig. 5B). Afterwards, the high abundance of *Methanosarcina* overlapped or more precisely mitigated this correlation. Finally and in contrast to the maize reactor systems where *Methanosaeta* became pre-dominant after stabilizing the NH_4_^+^-N concentration, *Methanosarcina* (TRF-627bp) prevailed in the sugar beet reactor at a NH_4_^+^-N concentration around 200 mg kg_FM_^−1^. In regard to the general high abundance of *Methanosarcina* in the sugar beet reactors, it has to be mentioned that the VFA concentration and the pH value were measured shortly before feeding; hence, it can be assumed that the rapid utilization of the easy degradable compounds in the sugar beet silage led to an increased VFA concentration, followed by a drop in the pH values shortly after feeding. Consequently, the higher occurrence of *Methanosarcina* compared with *Methanosaeta* might be explained by their lower sensitivity to a drop in pH due to their spherical form and thus higher volume-to-surface ratio as well as their growth in cell clusters, which altogether limit the intake of acetate in the archaeal cell (De Vrieze *et al*., 2012).

### Conclusions

To conclude, this study provided interesting insights into the highly dynamic variation of the microbial communities, which converted maize and sugar beet silage into methane-containing biogas. According to the chemical differences of the supplied feedstocks, both the bacterial and the archaeal communities showed a clear substrate adaptation resulting in a feedstock-specific kinetic of the biogas production. It was demonstrated that members from the phylum *Bacteroidetes* are mainly involved in the degradation of low molecular weight substances such as sugar, ethanol and acetate, the main compounds of the sugar beet silage. In contrast to this, the structural and functional broader phylum *Firmicutes* were found in a higher abundance in the maize reactors due to their capacity of degrading complex polymers, especially cellulose. For both investigated systems, it was further shown that *Methanosaeta*, an indicator for a good-performing process, is highly sensitive against sudden stress situations such as a strong decrease in the NH_4_^+^-N concentration or a drop in the pH value. In both cases, the overall process did not failed, but rather was compensated by members from the genera *Methanoculleus* and/or *Methanosarcina*. All in all, the results of this study showed that maize and sugar beet silage are suitable feedstocks for AD as the biogas production process resulted in an equal amount of methane-containing biogas although the biogas was produced by different, highly feedstock-adapted microbial communities. Nevertheless, it has to be considered, especially by the biogas plant operators, that the AD of sugar beet silage seems to be more susceptible to stress. Therefore, it is advisable to ensure a high structural and functional microbial diversity, especially when a new biogas plant is started.

## Experimental procedures

### Reactor construction and operation

Laboratory-scale CSTRs were used to investigate the adaptation, the efficiency and the dynamics of process relevant microorganisms during the AD of maize silage and sugar beet silage. For each feedstock, three parallel CSTRs with a working volume of 3 l were operated at mesophilic conditions (40°C). To ensure a high diversity of the starter-community, all reactors were inoculated with digestate from a mesophilic agricultural biogas plant operated with mixed manure, grass and maize silage. This digestate was diluted 1:2 with tap water before inoculation. The reactors were started with an OLR of 0.5 g_VS_ l^−1^ day^−1^ and slowly, after a minimum of 2 weeks, increased with 0.5 g_VS_ l^−1^ day^−1^ until a final OLR of 2 g_VS_ l^−1^ day^−1^ was reached (VDI, 2006). A correction of the TS for the calculation of the OLR was performed according to Weißbach and Strubelt (2008a; 2008b). To avoid process inhibition through lack of micronutrients, 10 μl gVS^−1^ of the trace elements solution DSMZ 144 were added to each feeding according to Elhussein and Weiland (2009). Differences in the volume flow were balanced with tap water to gain the same hydraulic retention time (HRT) in all reactors. After day 267, the NH_4_^+^-N concentration was kept at a stable level of around 450 mg l^−1^ in the maize reactors and 200 mg l^−1^ in the sugar beet reactors. To calculate the required amount of NH_4_^+^-N, the digestate was analysed twice a week, and the difference between the actual and the desired value was added as ammonium carbonate (powder) with the feeding the day after the analysis.

The amount of produced biogas was measured with a novel gas measurement system, which enables a high temporal resolution (Tölle and Huth, 2014). Additionally, the biogas was collected in gasbags to measure the gas composition twice a week using the portable analyser ‘Biogas Check’.

Following chemical measurements and analyses of the feedstock and reactor digestate were performed according to VDLUFA (1997): pH, TS, VS, VFAs in terms of acetate, propionate, iso- and n-butyrate, iso- and n-valerate and capronate, alcohols, TKN and NH_4_^+^-N. Additionally, neutral detergent fibre, acid detergent fibre and acid detergent lignin were determined in order to calculate the amount of cellulose, hemicellulose and lignin of the feedstock as well as sugar and starch (VDLUFA, 1997; Schönberg and Linke, 2012).

### Sampling and extraction of microbial DNA

During the start-up phase of the reactors, digestate samples were taken at the end of each OLR stage (day 33, 57 and 93). With achieving the final OLR, six further time points were selected for molecular biological analysis (day 141, 175, 232, 267, 309 and 337). The digestate samples were stored at −20°C until further analysis.

Total microbial genomic DNA was extracted using the PowerSoil® DNA Isolation Kit (MoBio Laboratories, USA). The DNA isolation was carried out according to the user’s manual except for the mechanical lysis, which was performed using the FastPrep® instrument (MP Biomedical, USA) for 2 × 20 s at 5 m s^−1^. For each sample, DNA from three subsamples was extracted. The extracted DNA was used as template for the TRFLP analyses as well as for the construction of 16S rRNA gene sequence libraries to characterize the diversity and dynamics of the bacterial and archaeal communities.

### Analyses of the microbial community dynamics by TRFLP

To detect the microbial community dynamics of the reactors, the fingerprint method TRFLP was used as previously described by Rademacher and colleagues (2012). For the amplification of the 16S rRNA gene, the bacterial primer pair 27F (5’-AGAGTTTGATCMTGGCTCAG-3’) and 926MRr (5’-CCGTCAATTCMTTTRAGTTT-3’) and the archaeal primer pair Ar109f (5′-ACKGCTCAGTAACACGT-3’) and Ar912r (5’-CTCCCCCGCCAATTCCTTTA-3’) were used. For both the bacterial and the archaeal amplifications, the forward primer was labelled with Indodicarbocyanine (Cy5) at the 5′-end. All used primers were provided by Biomers (Germany). After verification of the PCR reaction in a 1.2% agarose gel, the PCR products were purified using Nucleospin® Gel and PCR Clean-up kit by Machery Nagel (Germany). The concentration of the purified PCR products was measured using a NanoPhotometer (Implen, Germany). A total amount of 200 ng PCR product was digested for 16 h at 37°C with MspI and Hin6I for the bacterial assay and with AluI for the archaeal assay. All enzymes were provided by Thermo Fisher Scientific Bioscience, Fermentas (Germany). The digested fragments (TRFs) were separated using GenomeLab™ GeXP Genetic Analysis System (Beckman Coulter, Germany) as described in Rademacher and colleagues (2012) to obtain TRFLP profiles.

The further analysis and comparison of the TRFLP profiles was conducted using BioNumerics 7.1 (Applied Maths, Belgium). The TRFLP profiles of each sampling point were evaluated separately in the fingerprint curve-processing window. Peaks lower than 185 relative fluorescent units were considered as ‘background noise’. False positive peaks were sorted out using the bleed throw detection. In the comparison window, a band matching was performed with a position tolerance of 0.1%. The band matching of the detected TRFs was exported to MS Excel and normalized to relative values of the total fluorescence. The median values of nine technical replicates for each feedstock digestion experiment were calculated, reloaded as character types into BioNumerics and subsequently used to calculate the pairwise distances between two sampling points applying the unweighted pair group method with arithmetic mean (UPGMA) algorithm with Pearson correlation. Pairwise distances were given as percentage of dissimilarity between microbial community profiles indicating the rate of community change. By this approach, the dissimilarity can be influenced by the number as well as the relative abundance of the detected TRFs.

### Identification of detected TRFs by construction and screening of 16S rRNA gene sequence libraries

For the identification of the detected TRFs, a cloning/sequencing approach was applied. The PCR amplification of the 16S rRNA gene was conducted using the same primer pairs (in this case unlabelled) and PCR conditions as mentioned above. The PCR products were purified using the Nucleospin® Gel and PCR Clean-up kit by Machery Nagel (Germany). Cloning of 16S rRNA gene amplicons was performed according to Rademacher and colleagues (2012). DNA sequencing was conducted by GATC Biotech AG (Germany).

The obtained sequences were processed using the software package BioNumerics 7.1 (Applied Maths, Belgium). After a quality check of the sequences, a multiple alignment was applied using default settings for the Needleman–Wunsch algorithm with a clustw similarity calculation in combination with an UPGMA clustering using the Kimura-2-parameter correction. Based on this, alignment sequences were grouped into operational taxonomic units (OTUs) at 97% (*Bacteria*) and 99% (*Archaea*) sequence similarity required for the identification at the species level (Kim *et al*., 2011). All OTUs obtained in this study have been deposited to the European Molecular Biology Laboratory and are available under accession numbers LN624228-LN624323 (*Bacteria*) and LN624324-LN624341 (*Archaea*). Subsequently, OTUs were identified using the RDP Naïve Bayesian rRNA Classifier Version 2.6 (Wang *et al*., 2007). Reference sequences with a homology of at least 80%, which is required for the sequence correlation at the phylum level, (Talbot *et al*., 2008; Kim *et al*., 2011) were selected for a phylogenetic assignment of the defined OTUs from the microbial community. Additionally, the defined OTUs were cut virtually using the restriction digest tool of BioNumerics 7.1 to assign the detected TRFs of the reactor samples.

### Ecological indices

To gain a better understanding of the ecological role of the investigated microbial community, various ecological indices were applied, which mainly based on the microbial resource management concept (Verstraete *et al*., 2007; Marzorati *et al*., 2008; Read *et al*., 2011). The richness (R) was determined as the total number of detected TRFs. Additionally, we defined the Lorenz curve and the derived Gini coefficient for each sample, which is related to information about the community organization (Verstraete *et al*., 2007; Marzorati *et al*., 2008; Wittebolle *et al*., 2009). The higher the Gini coefficient, the more uneven is the community.

## Conflict of interest

None declared.

## Supporting Information

**Table S1.** Main chemical characteristics of the maize reactor and sugar beet reactor digestates. All values are given as mean values of the three reactors (biological replicates) including the standard deviation. **Table S2.** Characteristics of the bacterial and archaeal community structure in the analysed lab-scale biogas reactors fed with maize resp. sugar beet silage indicated by the number of detected TRFs and the community organisation expressed as Gini coefficient.
