# Supplementary material for: Dynamic variation of the microbial community structure during the long-time mono-fermentation of maize and sugar beet silage
Source: Microb Biotechnol. 2015 Feb 25;8(5):764–75. doi: 10.1111/1751-7915.12263 (PMC4554465; doi:10.1111/1751-7915.12263)
Supplement: Supplementary file 1 [file mbt20008-0764-sd1.doc]

Supplementary material

Table S1: Main chemical characteristics of the maize reactor and sugar beet reactor digestates. All values are given as mean values of the three reactors (biological replicates) including the standard deviation.

| Days of  operation | **Digestate of the maize reactors** | | | | |
| --- | --- | --- | --- | --- | --- |
|  | TS | VS | ƞ_VS_ | pH | VFA |
|  | [%FM] | [%TS] | [%] | [-] | [g L^-1^] |
| 0 | 3 | 74.4 | - | 7.6 | 0.56 |
| 33 | 2.6 ± 0.0 | 69.6 ± 0.1 | 90 ± 0.1 | 7.2 ± 0.0 | 0.0 ± 0.0 |
| 57 | 2.8 ± 0.1 | 71.2 ± 0.8 | 89 ± 0.6 | 7.3 ± 0.0 | 0.1 ± 0.0 |
| 93 | 3.0 ± 0.1 | 75.2 ± 0.4 | 87 ± 0.5 | 7.2 ± 0.0 | 0.1 ± 0.0 |
| 141 | 3.6 ± 0.1 | 75.1 ± 0.2 | 85 ± 0.5 | 7.1 ± 0.0 | 0.1 ± 0.0 |
| 175 | 4.0 ± 0.2 | 78.5 ± 1.0 | 82 ± 1.0 | 7.1 ± 0.1 | n.d. |
| 232 | 4.4 ± 0.2 | 80.0 ± 0.9 | 80 ± 0.9 | 7.1 ± 0.0 | 0.2 ± 0.0 |
| 267 | 4.4 ± 0.0 | 81.3 ± 0.8 | 80 ± 0.2 | 7.0 ± 0.0 | 0.0 ± 0.0 |
| 309 | 4.6 ± 0.3 | 82.8 ± 1.2 | 79 ± 1.8 | 7.0 ± 0.1 | 0.2 ± 0.0 |
| 337 | 4.6 ± 0.1 | 84.1 ± 0.2 | 78 ± 0.3 | 7.0 ± 0.0 | 0.1 ± 0.1 |
| Days of  operation | **Digestate of the sugar beet reactors** | | | | |
|  | TS | VS | ƞ_VS_ | pH | VFA |
|  | [%FM] | [%TS] | [%] | [-] | [g L^-1^] |
| 0 | 3 | 74.4 | - | 7.6 | 0.56 |
| 33 | 2.5 ± 0.1 | 68.0 ± 0.4 | 87 ± 0.2 | 7.3 ± 0.0 | 0.0 ± 0.0 |
| 57 | 2.5 ± 0.0 | 68.5 ± 0.6 | 87 ± 0.1 | 7.4 ± 0.0 | 0.2 ± 0.1 |
| 93 | 2.3 ± 0.0 | 69.2 ± 0.3 | 88 ± 0.2 | 7.3 ± 0.0 | 0.1 ± 0.0 |
| 141 | 2.6 ± 0.0 | 69.4 ± 1.2 | 87 ± 0.2 | 7.2 ± 0.0 | 0.1 ± 0.0 |
| 175 | 2.7 ± 0.0 | 68.7 ± 1.2 | 86 ± 0.2 | 7.2 ± 0.0 | n.d. |
| 232 | 2.7 ± 0.1 | 70.4 ± 2.0 | 86 ± 0.7 | 7.2 ± 0.1 | 0.2 ± 0.0 |
| 267 | 2.7 ± 0.1 | 68.1 ± 1.0 | 87 ± 0.4 | 7.2 ± 0.0 | 0.0 ± 0.0 |
| 309 | 2.5 ± 0.1 | 69.1 ± 1.2 | 87 ± 0.5 | 7.2 ± 0.0 | 0.2 ± 0.0 |
| 337 | 2.6 ± 0.0 | 70.5 ± 0.1 | 87 ± 0.2 | 7.2 ± 0.0 | 0.1 ± 0.0 |

TS = total solids, FM = fresh mass, VS = volatile solids, ƞ_VS_ = VS degradation degree, VFA = volatile fatty acids, n.d. = not determined.

Table S2: Characteristics of the bacterial and archaeal community structure in the analysed lab-scale biogas reactors fed with maize resp. sugar beet silage indicated by the number of detected TRFs and the community organisation expressed as Gini coefficient.

| Days of operation |  | **Richness (no. of TRFs)** | | | |  | **Community organization (Gini coefficient)** | | | |
| --- | --- | --- | --- | --- | --- | --- | --- | --- | --- | --- |
|  |  | **Bacteria** | | **Archaea** | |  | **Bacteria** | | **Archaea** | |
|  |  | Maize | Sugar beet | Maize | Sugar beet |  | Maize | Sugar beet | Maize | Sugar beet |
| Inoculum |  | 54 | 54 | 5 | 5 |  | 0.54 | 0.54 | 0.41 | 0.41 |
| 33 |  | 47 | 51 | 7 | 7 |  | 0.42 | 0.46 | 0.44 | 0.44 |
| 57 |  | 46 | 39 | 8 | 8 |  | 0.42 | 0.46 | 0.52 | 0.49 |
| 93 |  | 44 | 42 | 8 | 8 |  | 0.41 | 0.51 | 0.59 | 0.43 |
| 141 |  | 41 | 30 | 5 | 8 |  | 0.43 | 0.52 | 0.67 | 0.58 |
| 175 |  | 41 | 25 | 5 | 8 |  | 0.34 | 0.54 | 0.52 | 0.5 |
| 232 |  | 53 | 33 | 7 | 8 |  | 0.37 | 0.54 | 0.62 | 0.54 |
| 267 |  | 27 | 44 | 3 | 7 |  | 0.31 | 0.51 | 0.41 | 0.5 |
| 309 |  | 38 | 34 | 5 | 6 |  | 0.43 | 0.42 | 0.66 | 0.52 |
| 337 |  | 42 | 30 | 3 | 3 |  | 0.36 | 0.43 | 0.57 | 0.43 |
